# New Insights into the Potential Roles of 3-Iodothyronamine (T1AM) and Newly Developed Thyronamine-Like TAAR1 Agonists in Neuroprotection

**DOI:** 10.3389/fphar.2017.00905

**Published:** 2017-12-12

**Authors:** Lorenza Bellusci, Annunziatina Laurino, Martina Sabatini, Simona Sestito, Paola Lenzi, Laura Raimondi, Simona Rapposelli, Francesca Biagioni, Francesco Fornai, Alessandra Salvetti, Leonardo Rossi, Riccardo Zucchi, Grazia Chiellini

**Affiliations:** ^1^Laboratory of Biochemistry, Department of Pathology, University of Pisa, Pisa, Italy; ^2^Section of Pharmacology and Toxicology, Department of Psychology, Neurology, Drug Sciences, Health of the Child, Pharmacology, University of Florence, Florence, Italy; ^3^Laboratory of Medicinal Chemistry, Department of Pharmacy, University of Pisa, Pisa, Italy; ^4^Unit of Human Anatomy, Department of Translational Research and New Technologies in Medicine and Surgery, University of Pisa, Pisa, Italy; ^5^IRCCS Neuromed, Pozzilli, Italy; ^6^Unit of Experimental Biology and Genetics, Department of Clinical and Experimental Medicine, University of Pisa, Pisa, Italy

**Keywords:** thyronamines, trace amine-associated receptors, learning, memory, neuroprotection, autophagy, PI3K-AKT-mTOR

## Abstract

3-Iodothyronamine (T1AM) is an endogenous high-affinity ligand of the trace amine-associated receptor 1 (TAAR1), detected in mammals in many organs, including the brain. Recent evidence indicates that pharmacological TAAR1 activation may offer a novel therapeutic option for the treatment of a wide range of neuropsychiatric and metabolic disorders. To assess potential neuroprotection by TAAR1 agonists, in the present work, we initially investigated whether T1AM and its corresponding 3-methylbiaryl-methane analog SG-2 can improve learning and memory when systemically administered to mice at submicromolar doses, and whether these effects are modified under conditions of MAO inhibition by clorgyline. Our results revealed that when i.p. injected to mice, both T1AM and SG-2 produced memory-enhancing and hyperalgesic effects, while increasing ERK1/2 phosphorylation and expression of transcription factor *c*-fos. Notably, both compounds appeared to rely on the action of ubiquitous enzymes MAO to produce the corresponding oxidative metabolites that were then able to activate the histaminergic system. Since autophagy is key for neuronal plasticity, in a second line of experiments we explored whether T1AM and synthetic TAAR1 agonists SG1 and SG2 were able to induce autophagy in human glioblastoma cell lines (U-87MG). After treatment of U-87MG cells with 1 μM T1AM, SG-1, SG-2 transmission electron microscopy (TEM) and immunofluorescence (IF) showed a significant time-dependent increase of autophagy vacuoles and microtubule-associated protein 1 light chain 3 (LC3). Consistently, Western blot analysis revealed a significant increase of the LC3II/LC3I ratio, with T1AM and SG-1 being the most effective agents. A decreased level of the p62 protein was also observed after treatment with T1AM and SG-1, which confirms the efficacy of these compounds as autophagy inducers in U-87MG cells. In the process to dissect which pathway induces ATG, the effects of these compounds were evaluated on the PI3K-AKT-mTOR pathway. We found that 1 μM T1AM, SG-1 and SG-2 decreased pAKT/AKT ratio at 0.5 and 4 h after treatment, suggesting that autophagy is induced by inhibiting mTOR phosphorylation by PI3K-AKT-mTOR pathway. In conclusion, our study shows that T1AM and thyronamine-like derivatives SG-1 and SG-2 might represent valuable tools to therapeutically intervene with neurological disorders.

## Introduction

Trace amine-associated receptors (TAARs) represent a novel class of G protein coupled receptors (GPCRs) identified in 2001 by two independent groups (Borowsky et al., 2001; Bunzow et al., 2001). At first they were suggested to bind low abundance neurotransmitters termed “trace amines” (TAs), namely β-phenylethylamine, *p*-tyramine, tryptamine, and octopamine (Lindemann et al., 2005). The best-described member of the TAARs family is TAAR1 (Borowsky et al., 2001; Lindemann et al., 2005), which is relatively conserved among different animal species. TAAR1 is homologous to the dopamine, noradrenaline, and serotonin receptors and expressed in multiple organs including the brain, heart, liver, kidney, pancreas, and spleen. Although, its molecular signaling mechanisms have not yet been fully elucidated, TAAR1 couples to a Gα*s* protein and, upon stimulation, increases intracellular generation of *c*AMP via adenylyl cyclase activation, thus stimulating the inwardly rectifying K^+^ channels (Borowsky et al., 2001; Bunzow et al., 2001; Miller et al., 2005; Xie et al., 2007; Bradaia et al., 2009). TAAR1 signaling has been shown to promote PKA and PKC phosphorylation, resulting in the upregulation of CREB and NFAT transcription factors in activated lymphocytes (Panas et al., 2012), suggesting a possible role for TAAR1 in the modulation of the immune function. Besides Gαs-protein, TAAR1 signaling also involves G-protein independent pathways, such as the β-arrestin2-dependent pathway engaging the protein kinase B (AKT)/glycogen synthase kinase 3 (GSK3β) signaling cascade, a key player in dopaminergic neurotransmission (Harmeier et al., 2015).

**FIGURE 1:**
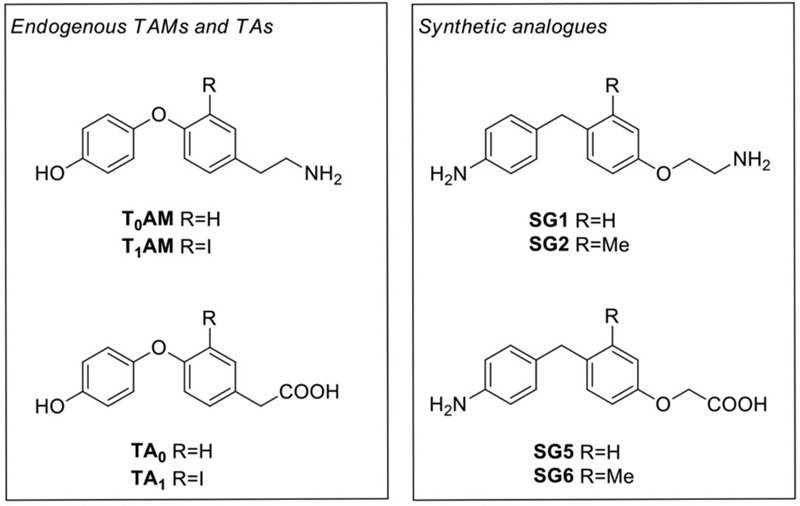
Chemical structures of thyronamines (T_0_AM, T_1_AM), their thyroacetic acid catabolites (TA_0_, TA_1_) and the corresponding synthetic analogs (SG1, SG2, SG5, and SG6)

During the past two decades, trace amine-associated receptor 1 (TAAR1) has been the focus of extensive research, particularly because, in addition to TAs as principal binding ligands, it is also activated by a number of endogenous and exogenous molecules, including catecholamines, amphetamine and amphetamine-like compounds, ergot derivatives and several adrenergic ligands (Bunzow et al., 2001; Pei et al., 2016), as well as certain recently discovered thyroid hormones derivatives, named thyronamines (TAMs) (Scanlan et al., 2004; Hart et al., 2006). Since 2004 two specific TAMs (**Figure 1**), namely 3-iodothyronamine (3-T_1_AM or T1AM) and thyronamine (T_0_AM), proposed to derive from thyroid hormone, TH, through deiodination and decarboxylation, were identified in various organs, including brain, liver, pancreas, and in human plasma, with T_1_AM being the most abundant (Scanlan et al., 2004; Scanlan, 2009; Saba et al., 2010; Zucchi et al., 2014; Hoefig et al., 2016; Chiellini et al., 2017). T1AM was considered as a novel chemical messenger since it was found to produce functional effects being a powerful and rapid activator of the rat and mouse TAAR1 (Scanlan et al., 2004; Scanlan, 2009).

Notably, the T1AM skeleton includes a β-phenylethylamine structure, a feature that may confer T1AM the ability of recognizing multiple cell targets including other G protein-coupled receptors, such as adrenergic receptors ADRα2A and ADRβ2 (Dinter et al., 2015a,b), muscarinic receptors (Laurino et al., 2016) and TAAR type 5 (TAAR5) (Dinter et al., 2015c) as well as ion channels, particularly the thermosensitive transient receptor potential melastatin 8 channel (TRPM8) (Khajavi et al., 2015). On the other hand, ligands of the dopamine, serotonin, histamine, or noradrenaline receptors also activate TAAR1-mediated signaling, although with a low potency (Borowsky et al., 2001; Bunzow et al., 2001; Alexander et al., 2013a). The co-existence of the aforementioned ligands in mammals brings into question the occurrence of cross-interrelations at the level of ligand–receptors interactions, which may impact at multiple levels orchestrated signaling events and physiological responses.

Pharmacological administration of T_1_AM affects reversibly and dose-dependently reversible effects on body temperature, cardiac function, energy metabolism, and neurological functions (Zucchi et al., 2014). Moreover, intracerebral administration of T1AM at doses close to its physiological levels (μg/kg) improves learning ability, reverses amnesia, modulates sleep and feeding, and reduces pain threshold to hot stimuli in mice (Manni et al., 2013; Laurino et al., 2017). Notably, these effects are prevented by pre-treatment with the monoamine-oxidase (MAO)-A inhibitor clorgyline (Alexander et al., 2013b), suggesting that the oxidative deamination of T1AM into 3-iodothyroacetic acid (TA1) (**Figure 1**) might contribute, at least in part, to the acute effects of T1AM *in vivo*. Recent findings provided compelling evidence that TA1 is generated *in vivo* by T1AM and that it plays a significant role in T1AM-induced behavioral effects (Laurino et al., 2015a,b). These observations strongly suggest the potential of T1AM and its metabolites/analogs for the treatment of endocrine and neurodegenerative disorders, including memory dysfunction (Manni et al., 2013; Laurino et al., 2015a). Recently, specific TAAR1 antagonists were used to acquire preliminary evidence that at least some electrophysiological effects of T1AM in the entorhinal cortex are mediated by TAAR1 (Accorroni and Zucchi, 2016).

Collectively, these findings indicated that T1AM might represent a valuable tool to investigate the physiological role and pharmacological potentials of TAAR1, as well as a good starting point to advance development of new targets for potential therapeutic interventions in a wide array of pathological processes, including metabolic, endocrine, and neurological disorders. In this context, with the aim to ameliorate the number of selective TAAR1 agonists, Chiellini et al. (2015, 2016), recently developed a novel class of halogen free biaryl-methane thyronamine analogs, namely SG compounds, synthetically more accessible than traditional thyronamines. Following a multidisciplinary approach, they identified two compounds, namely SG-1 and SG-2 (**Figure 1**), that appeared to be equipotent to endogenous thyronamines (i.e., T0AM and T1AM, respectively), providing additional tools for studying the physiological roles of TAAR1 receptor using *in vitro* and/or *in vivo* models.

To assess the therapeutic potential of thyronamine derivatives for neuroprotection, in the present paper, we initially investigated whether T1AM and its corresponding 3-methylbiaryl-methane analog SG-2 can improve learning and memory when systemically administered to mice at submicromolar doses, and whether these effects are modified under conditions of MAO inhibition by clorgyline. In addition, several lines of evidence indicate that autophagy (ATG), the process by which cells digest their own cytoplasmic constituents within lysosomes, is key for neuronal plasticity (Levine and Kroemer, 2008; Son et al., 2012). The dynamic process of autophagy, defined as autophagy flux, is orchestrated by several autophagy-related proteins, among which the yeast ATG-8 mammalian homolog microtubule-associated protein 1 light chain 3 (LC3) (mammalian homolog of yeast Atg8) is commonly used as an important biomarker of the autophagic process (Kabeya et al., 2000; Tanida, 2011; Sarkar, 2013). In addition to LC3, other autophagy substrates can be exploited for monitoring autophagy flux (Mizushima et al., 2010). Among these, p62, also known as SQSTM1/sequestosome 1, is perhaps the most studied. Indeed, p62 binds directly to LC3 and is selectively incorporated into autophagosomes, and subsequently degraded by autophagy (Bjørkøy et al., 2005), leading to an inverse correlation between total p62 cellular expression levels and autophagic activity.

Impairments of the autophagic process are associated with several neurodegenerative disorders, such as Alzheimer’s disease (AD), Parkinson’s disease, and Huntington’s disease, where a deficiency in the elimination of defective or aggregated proteins may lead to cellular stress, failure and death. As such, the induction of autophagy may be exploited as a strategy to assist neurons clearing abnormal protein aggregates and thus survive (Nixon, 2013). The kinase mammalian target of rapamycin (mTOR) is a major modulator of autophagy (Crino, 2016; Garza-Lombó and Gonsebatt, 2016). PI3K/AKT pathway modulates mTOR activity which is altered in neurodegenerative diseases, including Alzheimer’s and Parkinson’s disease (Heras-Sandoval et al., 2014). In particular, in dementia a marked up-regulation of mTOR, which relates with disease severity, has been described in humans (Tramutola et al., 2015). Hence, the ideal cell line for *in vitro* studies should feature a similar mTOR up-regulation. This is the case for U-87MG cells as we demonstrated in previous papers (Arcella et al., 2013; Ferrucci et al., 2017; Ryskalin et al., 2017) in which mTOR up-regulation leads to ATG suppression. Incidentally, the suppression of ATG in degenerative dementia is considered to be responsible also for altered glia-related inflammation. Thus, the U-87MG cell line is also well suited to investigate the involvement of glial cells since they represent an immortalized glioblastoma derived glial cells where mTOR upregulation is prominent. The ability to suppress mTOR in this cell line is remarkable due to an aberrant baseline mTOR overexpression just like in dementia (Wang et al., 2013).

Therefore, to explore the potential neuroprotective effects of thyronamines, we also evaluated the ability of T1AM and newly developed analogs SG-1 and SG-2 to promote autophagy in U-87MG cells, while investigating the signaling pathway being involved.

## Materials and Methods

### Chemicals

3-Iodothyronamine was kindly provided by Prof. Thomas Scanlan (Portland, OR, United States) and was dissolved in 0.5% DMSO (Veh). SG-1, SG-2, and SG-6 analogs were synthesized by our group according a procedure previously described (Chiellini et al., 2015) and were dissolved in 0.5% DMSO (Veh). Clorgyline and pyrilamine were supplied by Sigma–Aldrich, St. Louis, MO, United States.

### Animals

Experiments and animal care used procedures were in accordance with the National Institutes of Health Guide for the Care and Use of Laboratory Animals (NIH Publications No. 80-23, revised 1996). The experimental protocols were approved by the Animal Care Committee of the Department of Pharmacology, University of Florence, in compliance with the European Convention for the Protection of Vertebrate Animals used for Experimental and Other Scientific Purposes (ETS no. 123) and the European Communities Council Directive of 24 November 1986 (86/609/EEC). The authors further attest that all efforts were made to minimize the number of animals used and their suffering.

Male mice (CD1 strain; 20–30 g) from Envigo (Udine, Italy) were used. Five mice were housed per cage and the cages were placed in the experimental room 24 h before the test for adaptation. Animals were kept at 23 ± 1°C with a 12 h light–dark cycle (light on at 07:00 h) and were fed a standard laboratory diet with water *ad libitum*. All studies involving animals are reported in accordance with the ARRIVE guidelines for reporting experiments involving animals (Kilkenny et al., 2010; McGrath et al., 2010).

### Behavioral Tests

#### The Passive Avoidance Paradigm: The Light–Dark Box

The passive avoidance paradigm test using the light–dark box apparatus was carried out using the step-through method by Jarvik and Kopp (1967), and following a procedure already described in previous studies (Manni et al., 2013; Musilli et al., 2014). The test was carried out by comparing the effect of vehicle (Veh; 0.5% DMSO) or test compound (i.e., T1AM or SG-2; 1.32, 4, and 11 μg⋅kg^-1^; *n* = 20 for each treatment) injected i.p. to the mice prior to the training session of the test.

#### Determination of Mice Pain Threshold to Thermal Stimulus Using the Hot Plate

Mice were injected i.p. with test compound (i.e., T1AM or SG-2; 1.32, 4, and 11 μg⋅kg^-1^) or vehicle (*n* = 20 for each group) and, after 15 min, they were placed on the hot plate set at 51.5°C. The time taken to observe a licking, flinching or jumping response was measured. The cut-off time was set at 45 s to minimize skin damage.

In another set of experiments, additional mice were pretreated with i.p. clorgyline (2.5 mg⋅kg^-1^) or with the histamine H1 receptor antagonist pyrilamine (10 mg⋅kg^-1^) and after 30 min they received the test compound (i.e., T1AM or SG-2; 1.32, 4, and 4 μg⋅kg^-1^) or saline (i.p.). Measurements were performed 15 min after i.p. injections.

#### Identification of T1AM or SG-2 Activated Signaling in Mouse Brain and Dorsal Root Ganglion (DRG)

##### Mouse brain and DRG tissue preparations

Mice were removed from the cage and injected i.p. with vehicle or test compound (i.e., T1AM or SG-2; 1.32, 4, and 11 μg⋅kg^-1^).

Animals were sacrificed at two different time points, 15 min or 5 h, after test compound or vehicle administration. The brain was excised and the forebrain was split into two sections: frontal cortex and diencephalon. The hippocampus, hypothalamus/thalamus were then quickly removed, and all tissues were flash-frozen in liquid nitrogen and stored at -80°C until use. In another set of experiments, mice were sacrificed 15 min after i.p. injection with vehicle or test compound (i.e., T1AM or SG-2; 1.32, 4, and 11 μg⋅kg^-1^). DRG were freshly isolated using a previously described procedure (Vellani et al., 2004). Briefly, 20–30 ganglia were isolated from the full length of the spinal column following removal of the spinal cord, and stored in 0.5% SDS at -80°C until use.

Brain and DRG samples were homogenized in homogenization buffer containing (in mM): 50 Tris-HCl at pH 7.5, 150 NaCl, 1 EDTA, 5 sodium pyrophosphate, 10 β-glycerophosphate, 1 Na_3_VO_4_, 0.2 PMSF, 25 μg⋅mL^-1^ leupeptin, 10 μg⋅mL^-1^ aprotinin and 0.1% SDS. To remove cell debris, homogenates were centrifuged at 1000 × *g* for 10 min at 4°C and the supernatants (brain lysates) used for Western blot analysis.

##### Western blot analysis

Proteins (20 μg) isolated from selected brain regions and DRG homogenates were separated on 4–20% SDS-PAGE and transferred into PVDF membranes (60 min at 398 mA) using standard procedures. Membranes were blocked in PBST (PBS containing 0.1% Tween) containing 5% non-fat dry milk for 60 min. Blots were incubated overnight at 4°C with specific antibody against ERK1/2 phosphorylated on Thr^202^/Tyr^204^ (pERK1/2, Cell Signaling Technology) or c-fos (Sigma–Aldrich S.r.l, Milan, Italy). Primary antibodies were diluted in PTBS containing 3% albumin. After being washed with PBST, the membranes were incubated with polyclonal goat anti-rabbit HRP-conjugated secondary antisera (1:2,000, diluted in PTBS containing 5% non-fat dry milk) and left for 1 h at room temperature. Blots were then extensively washed and developed using an enhanced chemiluminescence detection system (Pierce Scientific, Rockford, IL, United States). Exposition and developing time were standardized for all blots. Densitometric analysis of scanned images was performed on a Macintosh iMac computer using the public domain NIH Image program. Glyceraldehyde-3-phosphate dehydrogenase (GAPDH, Sigma–Aldrich S.r.l., Milan, Italy) or β-actin (Sigma–Aldrich, S.r.l., Milan, Italy) were used as loading control. Protein concentration was quantified using Bradford’s method (protein assay kit, Bio-Rad Laboratories, Segrate, Milan, Italy).

Data represent mean ± SEM of three different experiments. Results are expressed as arbitrary units (AU), consisting of the ratio between the expression levels of the protein of interest and that of the GAPDH.

### Induction of Autophagy in Cell Lines

#### Cell Lines

U-87MG human glioma cell lines from Cell Bank (IRCC San Martino-IST, Genova) were cultured in standard DMEM-High Glucose medium (Sigma–Aldrich S.r.l., Milan, Italy) supplemented with 10% fetal bovine serum (FBS), 1% of MEM non-essential amino-acid (MEM-NEAA), penicillin (50 IU/mL) and 100 μg streptomycin (Sigma–Aldrich S.r.l., Milan, Italy). Cells were kept at 37°C in a humidified atmosphere with 5% CO_2_ and the medium was renewed two to three times per week.

For transmission electron microscopy (TEM) and Western blot (WB) assays, the cells were cultured at a density of 1 × 10^6^ cells/well in a 6-well plate in a final volume of 2 ml/well. For confocal light microscopy (IF) assay 3 × 10^4^ cells were seeded on cover- slips in 24-well plates in a final volume of 1 ml/well. In order to evaluate cytotoxicity (Trypan blue assay) and cell viability (MTT assay), 1 × 10^4^ cells were seeded in 96-well plate in a final volume of 400 ml/well. Twenty-four hours after seeding, the cells were treated with test compounds (T1AM, SG1, and SG2) at the dose of 1 μM for different exposure times (30’, 4, 8, and 24 h). Dilutions of test compounds were obtained by a stock solution (1 mM in saline containing 10% DMSO).

#### Trypan Blue (Cytotoxicity Assay)

For trypan blue staining, after incubation with the different drugs the cells were collected and centrifuged at 800 rpm for 5 min. The cell pellet was suspended in culture medium and 25 μl of the cellular suspension were added to a solution containing 1% of trypan blue (62.5 μl) and PBS (37.5 μl). The cells were incubated for 5 min at room temperature. Then, a volume of 10 μl of this solution was counted using a Bürker glass chamber and a light microscope. Viable and non-viable cells were recorded and the cell viability was expressed as a percentage of number of viable cells/number of total cells. The values represent the means of three independent cell counts.

#### MTT (Cell Viability Assay)

Cell viability was measured by using 3-(4,5-dimethylthiazol-2-yl)-2,5-diphenyltetrazolium bromide (MTT) reagent (Mosmann, 1983). In brief, after incubation with the different drugs, 10 μL of MTT reagent (0.5 mg/ml) was added to the medium and the cells were incubated for 3 h at 37°C. The resultant insoluble MTT-formazan dark red crystals were dissolved with 100 μl of solubilization buffer (10% SDS in 0.01M HCl) and after incubating for 7 h at 37°C, absorbance at OD570 nm was determined with an automated microplate reader (BIO-TEK, Winooski, VT, United States). The percentage of cell viability was calculated using the following formula: Cell viability (%) = Cells number (treated)/Cells number (DMSO control) Å ∼ 100.

#### Transmission Electron Microscopy (TEM)

Glioblastoma U-87MG cells were collected using trypsin and centrifuged at 1,000 × *g* for 5 min. After the supernatant was removed, the pellet was thoroughly rinsed in PBS. Cell pellets were fixed in 2.0% paraformaldehyde + 0.1% glutaraldehyde in 0.1M PBS (pH 7.4) for 90 min at 4°C. This procedure is used to prevent specific TEM artifacts caused by high concentrations of aldehydes, which could hinder our ability to detect authentic autophagy-like vacuoles (Fornai et al., 2001). Postfixation was done in an aqueous solution of 1% OsO_4_ for 1 h at 4°C; then the specimens were dehydrated in ethanol and embedded in Epoxy-resin. Ultrathin sections (50–70 nm) were cut using a Leica Ultramicrotome R and contrasted using uranyl acetate (saturated solution in distilled water) and lead citrate to be finally observed by using a Jeol JEM SX 100 electron microscope (Jeol).

For ultrastructural morphometry, non-serial sections were examined directly by TEM at 5,000× magnification. At least 10 cells per section were identified in each grid and several grids were examined to achieve a total of at least 30 cells per experimental group. Autophagy-like vacuoles, identified as double or multiple membranes (autophagosomes-like) containing cytoplasmic material and electrondense membranous structures (Fornai et al., 2007; Lenzi et al., 2012), were counted.

Values were calculated using ImageJ software and expressed as density of autophagy-like vacuoles (number of autophagy-like vacuoles per surface unit).

#### Confocal Microscopy (IF)

U-87MG cells grown overnight on coverslips were washed twice with phosphate-buffered saline (PBS) and fixed with methanol at room temperature for 5 min. Retrieval of the antigen was achieved in 100 μM Tris-HCl, 5% urea at 95°C for 10 min. After washing with PBS, cells were permeabilized with 0.2% Triton X-100 for 10 min at room temperature, and subsequently blocked in PBST (PBS + 0.1% Tween-20), supplemented with 1% bovine serum albumin (BSA) and 22.52 mg/ml of glycine, for 30 min. The cells were then incubated overnight at 4°C in 1% BSA in PBST containing 1:50 anti-LC3 antibody (Abcam, Cambridge, United Kingdom). After extensive PBST washes, cells were incubated for 1 h at room temperature with a 1:200 dilution of goat anti-rabbit secondary antibody (Alexa 488, Molecular Probes, Life Technologies) in 1% BSA in PBST. Cells were then washed with PBS, mounted in Prolong Diamond Antifade Mountant (Molecular Probes, Life Technologies) and subsequently examined. The analysis was performed using a Leica TCS SP5 confocal laser-scanning microscope (Leica Microsystems, Mannheim, Germany) using a sequential scan procedure. Confocal images were collected every 400 nm intervals through the *z*-axis of sections by means of 20^x^ and 63^x^ oil lenses. Z-stacks of serial optical planes were analyzed using the Multicolor Package software (Leica Microsystems). Negative controls were performed, omitting the primary antibody. Fluorescence intensity was analyzed by using ImageJ program.

#### Western Blot Analysis

1 × 10^6^ U-87MG cells were seeded in 6-well plates in a final volume of 2 ml/well and grown to 80% of confluence with standard medium (DMEM-High Glucose). Cells were treated with vehicle (0.1% DMSO) or 1 μM test compounds (T1AM, SG-1, and SG-2) and incubated at 37°C for 30 min, 4, 8, and 24 h.

Treated cells were washed twice with PBS and lysed in Tris-buffered saline buffer-1% Triton-X100; NaCl 150 mM; Tris-HCl 20 mM; EDTA 1 mM; EGTA 1 mM; NaF 20 mM; Na_4_P_2_O_7_ 25 mM; Na_3_VO_4_ 1 mM; PMSF 1 mM; 8 μl/ml protein cocktail inhibitors (Sigma–Aldrich, Milan, Italy). Proteins (20–30 μg) were separated on Criterion TGX^TM^ gel (4–20%) and transferred on Immuno-PVDF membrane (Bio-Rad, Milan, Italy) for 1 h. Blots were incubated for 12 h with diluted primary antibody [1:1000, LC3A/B; p62; Akt, p-Akt(Ser 473), β-actin, Cell Signaling] in 5% w/v BSA, 1X TBS and 0.1% Tween^®^ 20 at 4°C under gentle shaking. Then, blots were washed three times for 10 min with 1X TBS, 0.1% Tween^®^ 20 and incubated for 1 h with secondary antibody (peroxidase-coupled anti rabbit in 1X TBS, 0.1% Tween^®^ 20). After washing three times for 10 min, the reactive signals were revealed by enhanced ECL Western Blotting analysis system (Amersham). Band densitometric analysis was performed using Image Lab Software (Bio-Rad, Milan, Italy).

### Statistical Analysis

Data are reported as the mean ± SEM. Statistical analysis was performed by one-way analysis of variance (ANOVA), followed by Student–Newman–Keuls multiple comparison *post hoc* test. The threshold of statistical significance was set at *P* < 0.05. Data analysis was performed by Graph-Pad Prism 6.0 statistical program (GraphPad Software Inc., San Diego, CA, United States).

## Results

### T1AM and SG-2 Increase Memory Retention

3-Iodothyronamine is known to induce pro-learning and anti-amnestic responses when administered i.c.v. at very low doses (1.32–4 μg⋅kg^-1^) to mice, but its effects on memory after systemic administration are currently unknown. To address this point, we first evaluated the behavior of CD-1 mice injected i.p. with T1AM or its 3-methylbiaryl-methane analog SG-2 (1.32, 4, and 11 μg⋅kg^-1^) in the passive avoidance test.

**FIGURE 2:**
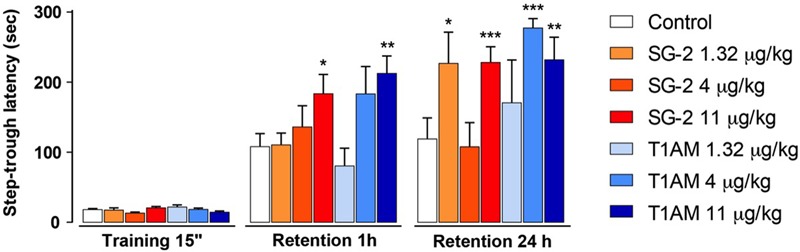
SG-2 and T1AM modified learning in mice. Mice were injected i.p. either with SG-2 (1.32, 4, or 11 mg⋅kg^-1^), T1AM (1.32, 4, or 11 mg⋅kg^-1^) or with vehicle (Control) and subjected to the passive avoidance test as described in the “Materials and Methods” section. Results are expressed as mean ± SEM; *n* = 20 in each group; ^∗^*P* < 0.05; ^∗∗^*P* < 0.01; ^∗∗∗^*P* < 0.001 versus vehicle.

As described in the “Materials and Methods” section, retention sessions of the passive avoidance test were performed 1 and 24 h after T1AM or SG-2 injection. In the 1 h retention session, following administration of 4 μg⋅kg^-1^ T1AM or SG-2 the latency to enter the dark compartment was increased, but the difference versus the control group did not reach the threshold of statistical significance. However, when either of the two compounds was administered at the dose of 11 μg⋅kg^-1^, a significantly higher latency to enter the dark compartment was observed at 1 h (*P* < 0.05 and *P* < 0.01 vs. vehicle-treated mice, respectively) (**Figure 2**). In the 24 h section both compounds appeared to show activity at lower doses, even though their effects did not follow a linear dose–response trend (**Figure 2**).

### T1AM and SG-2 Increase Pain Sensitivity

**FIGURE 3:**
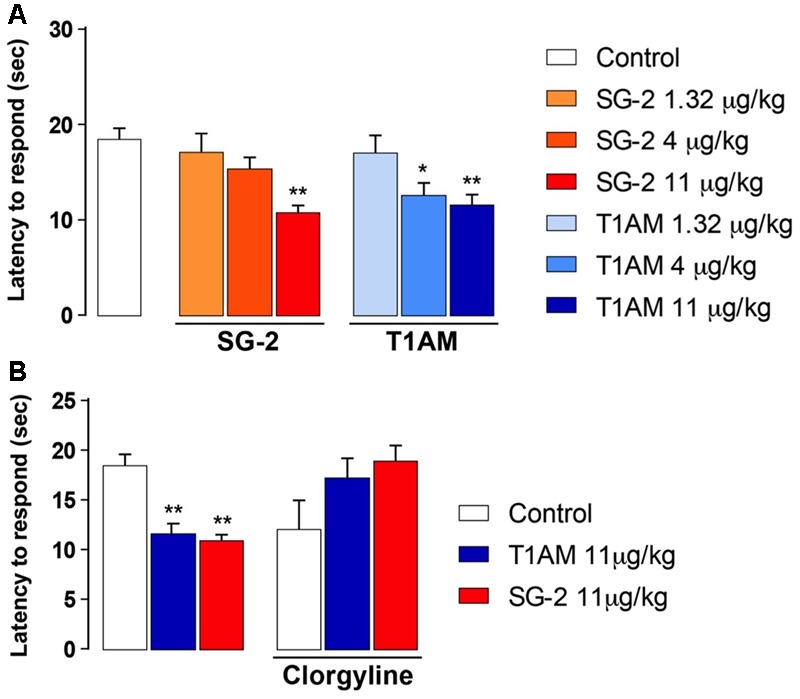
SG-2 and T1AM reduce nociceptive threshold in mice and the effect is blunted by clorgyline pretreatment. **(A)** Mice were placed on the hot plate 15 min after i.p. injection of either SG-2 (1.32, 4, or 11 mg⋅kg^-1^), T1AM (1.32, 4, or 11 mg⋅kg^-1^) or vehicle (Control). The latency to respond to the insult was evaluated as described in the “Materials and Methods” section. Results are expressed as mean ± SEM; *n* = 20 in each group; ^∗^*P* < 0.05 and ^∗∗^*P* < 0.01 versus vehicle. **(B)** Pain threshold was measured in mice treated with vehicle (Control), SG-2 or T1AM at their highest dosage (11 mg⋅kg^-1^). Experiments were repeated in mice pretreated with clorgyline (2.5 mg⋅kg^-1^, i.p.) 30 min before receiving the test drugs at the dosage of 11 mg⋅kg^-1^, or vehicle (Control). The nociceptive threshold was determined by the hot plate test. Results are expressed as mean ± SEM; *n* = 10 in each group; ^∗∗^*P* < 0.01 versus vehicle without clorgyline (Control, left panel).

Since the effect on memory enhancement that was observed in the passive avoidance test following i.p. injection of T1AM or SG-2 may involve an analgesic action, we next checked whether T1AM and SG-2 had any effect on pain threshold. As shown in **Figure 3A**, the hot-plate analgesic test revealed that, when injected i.p. to mice at doses that were effective in the passive avoidance test (i.e., 4 and 11 μg⋅kg^-1^ for T1AM, and 11 μg⋅kg^-1^ for SG-2), both compounds significantly reduced the pain perception threshold to hot insults (*P* < 0.01 and *P* < 0.05 vs. vehicle, respectively). Therefore, neither T1AM nor SG-2 showed any analgesic effect at the doses applied.

### Clorgyline Pretreatment Modulates the Response to T1AM and SG-2

Oxidative deamination by amine oxidases, followed by aldehyde oxidation by the widely distributed enzyme aldehyde dehydrogenase, is known to be the main metabolic pathway for T1AM (Wood et al., 2009; Saba et al., 2010), leading to the production of its metabolite 3-iodothyroacetic acid (TA1), which is not a ligand of TAAR1 (Chiellini et al., 2015). Notably, many of the neurological effects observed after administering T1AM i.c.v. were prevented or reduced in animals pre-treated with clorgyline, a MAO-A inhibitor (Manni et al., 2012, 2013), suggesting that TA1 may directly or indirectly contribute to the pharmacology of T1AM.

In addition, the pharmacokinetics of thyronamine-like synthetic analogs, such as SG-2, has not yet been completely described. Therefore, it seemed interesting to investigate whether the hyperalgesic effect observed after systemic administration of both T1AM and SG-2 was affected by the MAO inhibitor clorgyline, administered i.p. at the dose of 2.5 mg⋅kg^-1^.

In agreement with previous findings, the results of our pain threshold experiments revealed that the hyperalgesic effect produced by 11 μg⋅kg^-1^ T1AM or SG-2 was lost with clorgyline pretreatment (**Figure 3B**). Notably, in control mice i.p. injected with clorgyline an increased pain sensitivity was observed as compared to vehicle treated animals. In fact, as shown in **Figure 3B**, after treatment with i.p. clorgyline the observed latencies were generally lower than those observed in vehicle treated animals.

### Effects of H_1_ Antagonist Pyrilamine on the Hyperalgesic Response to T1AM and SG-2

Recent studies showed that i.c.v. injection in mice of equimolar doses of TA1 reproduced the pro-learning effects induced by T1AM. Notably, TA1 as well as T1AM pro-learning effects were modulated by histaminergic antagonists (Musilli et al., 2014; Laurino et al., 2015b). Therefore, it appeared interesting to further investigate whether the hyperalgesic effects observed in mice after 11 μg⋅kg^-1^ T1AM or SG-2 i.p. injection are mediated by the histaminergic system.

**FIGURE 4:**
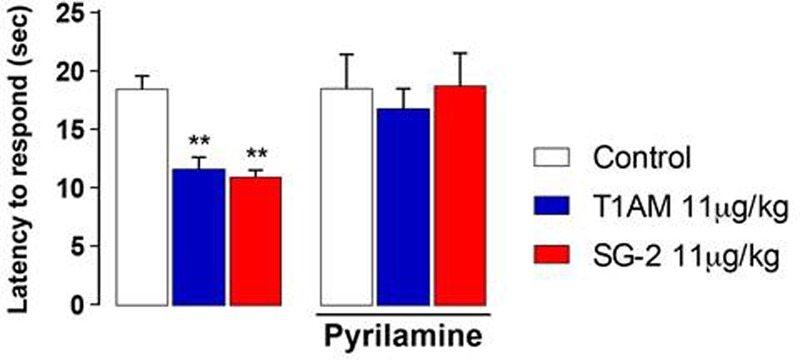
Pretreatment with H_1_ receptor antagonist pyrilamine abolishes SG-2 or T1AM-induced hyperalgesia. The nociceptive threshold of mice pre-treated (s.c.) with the H_1_ receptor antagonist pyrilamine (10 mg kg^-1^) was determined by the hot plate test 15 min after SG-2 or T1AM i.p. injection (11 μg kg^-1^) (*n* = 10 in each group). Results are expressed as means ± SEM; ^∗∗^*P* < 0.01 versus vehicle without pyrilamine (Control, left panel).

As shown in **Figure 4**, pretreatment with pyrilamine, a type1 histaminergic receptor (H1) antagonist, completely prevented the hyperalgesic effects of both T1AM and SG-2.

**FIGURE 5:**
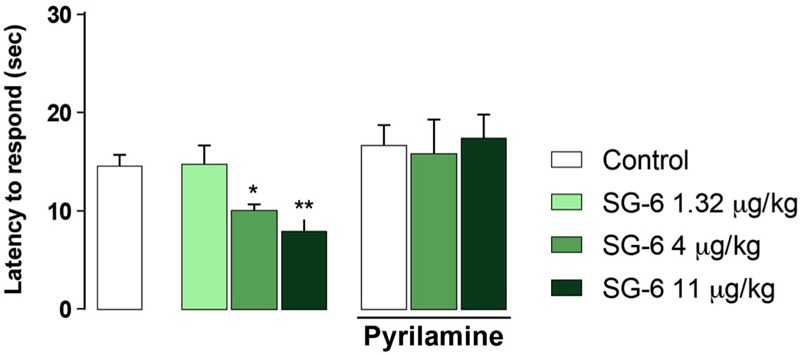
SG-6 reduces nociceptive threshold in mice and the effect is blunted by pyrilamine pretreatment. Mice were injected i.p. with SG-6 (1.32, 4, or 11 μg⋅kg^-1^) or with vehicle (Control) and after 15 min their nociceptive threshold measured by the hot plate test. Experiments were repeated in mice pre-treated (s.c.) with the H_1_ receptor antagonist pyrilamine (10 mg kg^-1^) and exposed to the hot plate test 15 min after SG-6 i.p. injection (4 or 11 μg kg^-1^). Results are expressed as means ± SEM; *n* = 10 in each group; ^∗^*P* < 0.05 and ^∗∗^*P* < 0.01 versus vehicle without pyrilamine (Control, left panel).

In addition, i.p. injection in mice of equimolar doses of SG-6 (**Figure 1**), the potential oxidative deamination derivative of SG-2, which essentially can be considered as the 3-methylbiaryl-methane analog of TA1, reproduced the hyperalgesic effects induced by SG-2. In agreement with previous findings on TA1, SG-6 effects were completely abolished by pretreatment with pyrilamine (**Figure 5**).

Notably, pyrilamine *per se* did not modify the pain perception to hot insults, which was found to be comparable to that of vehicle treated animals (**Figures 4, 5**).

### Systemic Administration of T1AM and SG-2 Activate Central Nervous System Signaling

**FIGURE 6:**
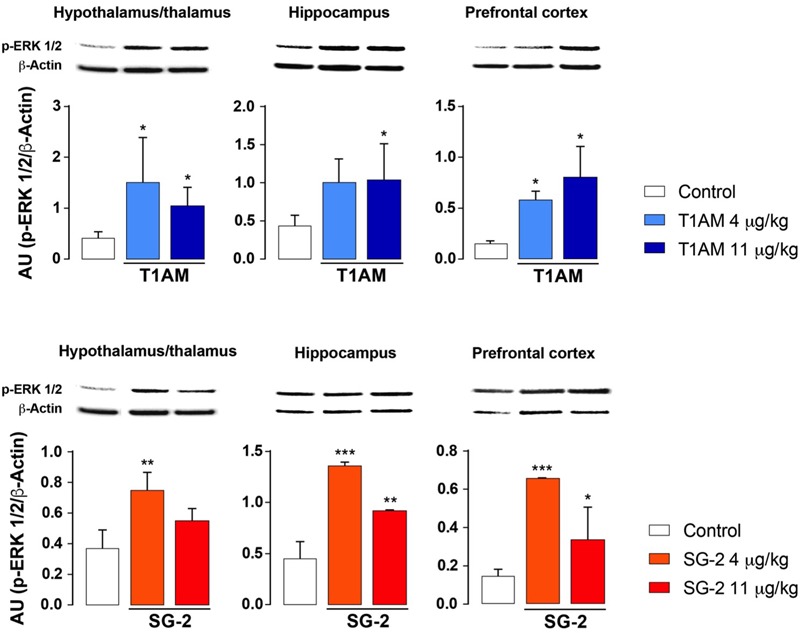
T1AM and SG-2 induce p-ERK in CD-1 mice brain regions. Fifteen minutes after i.p. injection of either T1AM, SG-2 (4, 11 mg⋅kg^-1^) or vehicle (Control), mice (*n* = 3 per group) were killed and brain regions separated as described in the “Materials and Methods” section. Immunodetection for p-ERK was carried out on protein lysates from each region separated on SDS-PAGE gels. A representative experiment for each brain region analyzed is shown. Results are the mean ± SEM of the densitometry of three different gels. ^∗^*P* < 0.05; ^∗∗^*P* < 0.01; ^∗∗∗^*P* < 0.001 versus vehicle.

**FIGURE 7:**
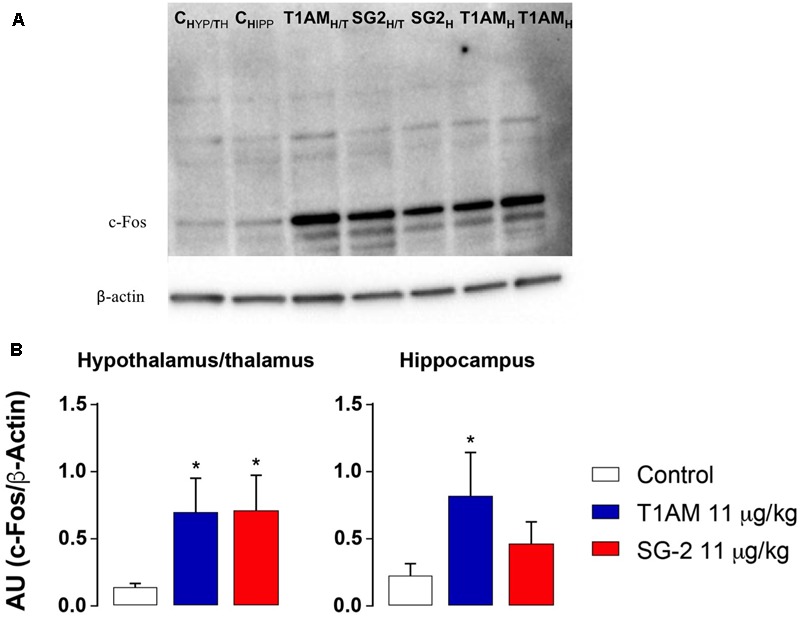
T1AM and SG-2 induce *c-fos* in CD-1 mice brain regions. Fifteen minutes after i.p. injection of either T1AM, SG-2 (11 mg⋅kg^-1^) or vehicle (Control), mice (*n* = 3 per group) were killed and brain regions separated as described in the “Materials and Methods” section. **(A)** Immunodetection for c-fos was carried out on protein lysates from each region separated on SDS-PAGE gels. A representative experiment is shown. **(B)** Western blot quantification of *c-fos* in mice brain regions after treatment with 11 mg⋅kg^-1^ T1AM or SG-2. Results are the mean ± SEM of the densitometry of three different gels. ^∗^*P* < 0.05 versus vehicle.

Increased ERK1/2 phosphorylation and increased expression of transcription factor *c-fos* are commonly associated to memory acquisition and storage (Giese and Mizuno, 2013; Minatohara et al., 2015). Indeed, Western blot analysis of selected brain region lysates (i.e., hypothalamus/thalamus, hippocampus, and prefrontal cortex) obtained from mice after treatment with T1AM or SG-2 at doses that demonstrated to be effective on memory acquisition and retention (i.e., 4 and 11 mg⋅kg^-1^) showed significantly increased pERK (^∗^*P* < 0.05; ^∗∗^*P* < 0.01; ^∗∗∗^*P* < 0.001 versus vehicle), and *c-fos* (^∗^*P* < 0.05 versus vehicle) expression as compared to control mice (**Figures 6, 7**).

**FIGURE 8:**
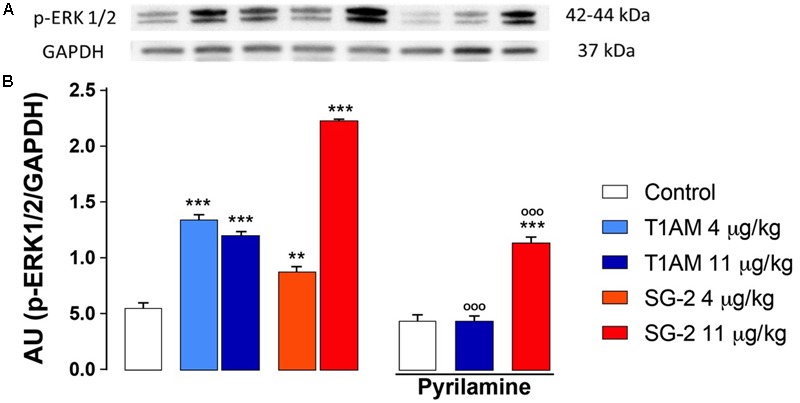
T1AM and SG-2 induce p-ERK1/2 in dorsal root ganglia (DRG) from CD1 mice: the effect of pyrilamine pretreatment. DRG were isolated from mice randomly treated with saline (Control), T1AM (4, 11 μg kg^-1^) or SG-2 (4, 11 μg kg^-1^) with or without being exposed to pretreatment with pyrilamine (10 mg kg^-1^) and analyzed for p-ERK1/2 levels by Western blot as described in Methods. **(A)** p-ERK1/2 in DRG protein lysates. A representative experiment is shown. **(B)** Densitometric analysis of p-ERK1/2 levels in DRG from CD1 mice. Results represent mean ± SEM of three different gels. ^∗∗^*P* < 0.01 versus vehicle (Control); ^∗∗∗^*P* < 0.001 versus vehicle (Control); ^∗∗∗^*P* < 0.001 versus T1AM 11 μg kg^-1^ or SG-2 11 μg kg^-1^.

Spinal extracellular signal-regulated kinases (ERKs) have long been known as mediators of nociceptive plasticity (Watkins et al., 1997; Wiertelak et al., 1997), and the measurement of pERK1/2 levels has recently emerged as an extremely reliable marker for neuronal activation and central sensitization after noxious stimulation and tissue injury (Gao and Ji, 2009). Therefore, we investigated the effects of systemic administration of T1AM or SG-2 on ERK1/2 activation in our experimental model. As depicted in **Figure 8**, in DRG prepared from mice treated with 4 and 11 μg⋅kg^-1^ T1AM or SG-2 a strong activation of ERK1/2 was observed (^∗∗^*P* < 0.01 versus vehicle; ^∗∗∗^*P* < 0.001 versus vehicle). Notably, in the same set of experiments, pretreatment with pyrilamine followed by administration of T1AM or SG-2 at the highest dosage significantly reduced pERK1/2 detection (^∗∗∗^*P* < 0.001 versus vehicle (Control); ^∗∗∗^*P* < 0.001 versus T1AM 11 μg kg^-1^ or SG-2 11 μg kg^-1^) (**Figure 8**).

### Induction of Autophagy in U-87MG Cells

Autophagy is a complex cellular lysosome-mediated process that eliminates misfolded proteins, protein complexes, or organelles through lysosomial degradation, and it is required for cellular homeostasis in cell survival, with neuronal cells representing one of the most studied systems (Mariño et al., 2011; Radad et al., 2015). An altered autophagy is described in several models of neurodegeneration, including Alzheimer’s and Parkinson’s diseases, as well as after traumatic and ischemic brain injuries (Galluzzi et al., 2016; Rivero-Rios et al., 2016; Li et al., 2017; Martinez-Vicente, 2017). Thus, stimulation of autophagy may lead to a new approach in the treatment of neurological diseases.

With the aim to explore the role played by T1AM and recently developed thyronamine-analogs SG-1 and SG-2 in the autophagy process, we examined the formation of autophagosomes and the autophagic flux in human glioblastoma cell lines (U-87MG). U-87MG cells are commonly used as a model to study neurological diseases, characterized by an up-regulation of mTOR (molecular target of rapamycin), an important inhibitor of autophagy (Arcella et al., 2013; Ryskalin et al., 2017). In our study, U-87MG cells were treated with 1 μM T1AM, SG-1 and SG-2 for 30 min, 4, 8, and 24 h and the progress of the autophagic flux was investigated both morphologically, by using TEM and immunofluorescence microscopy (IF), and biochemically, by monitoring the expression of autophagy marker proteins, such as LC3-II (Mizushima et al., 2010) and p62 (Bjørkøy et al., 2005), by Western blot analysis.

We then proceeded to elucidate which pathway was involved in ATG modulation by assessing our test compounds on the PI3K/AKT/mTOR signaling pathway (Song et al., 2005; Jung et al., 2010).

### Ultrastructural Studies

**FIGURE 9:**
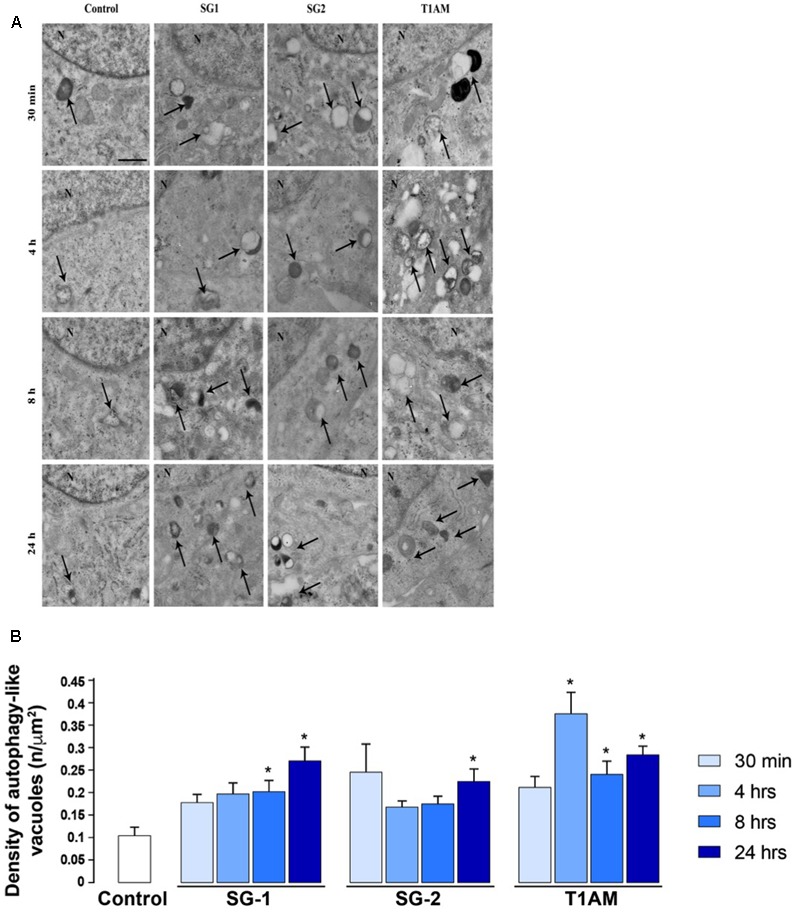
Transmission Electron Microscopy (TEM) shows time-dependent induction of autophagy in U-87MG cells by 1 μM SG-1, SG-2, or T1AM. **(A)** Representative pictures of ATG-like vacuoles (arrows) in the cytoplasm in baseline conditions (Control) and after treatment with 1 μM SG-1, SG-2, or T1AM, as visualized in representative micrographs. **(B)** The mean number of ATG-like vacuoles per cell was counted and values were reported in graph. Values are given as the mean ± SEM. Comparisons between groups were made by using one-way ANOVA. ^∗^*P* < 0.05 compared with baseline conditions. Scale bars **(A,B)** = 1 μm.

As shown in representative pictures reported in **Figure 9A** and in the graphs of **Figure 9B** the number of ATG-like vacuoles counted by plain TEM increased in U-87MG cells exposed to 1 μM T1AM, SG-1, or SG-2 for 0.5, 4, 8, and 24 h as compared to cells in baseline conditions. In detail, the number of vacuoles significantly increased after treatment with 1 μM T1AM for 4, 8, and 24 h (^∗^*P* < 0.05 compared with baseline conditions), whereas with 1 μM SG-1 or SG-2 significant effects were observed only after 8–24 h (^∗^*P* < 0.05 compared with baseline conditions), and 24 h treatment (^∗^*P* < 0.05 compared with baseline conditions), respectively (**Figure 9B**).

**FIGURE 10:**
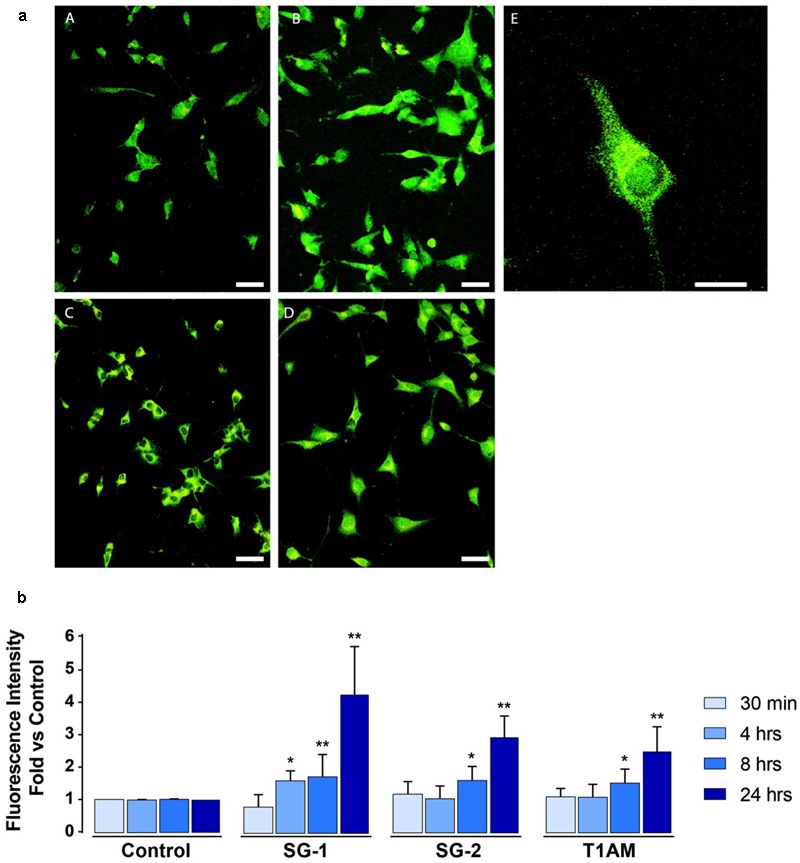
Confocal microscopy confirms induction of autophagy in U-87MG cells treated with 1 μM SG-1, SG-2, or T1AM. **(a)** Representative images of LC3-expressing cells incubated with 1 μM SG1, SG2 or T1AM 24 h after the treatment. (A) Control cells, (B) SG1-treated cells, (C) SG2-treated cells, (D) T1AM-treated cells. Scale bars are 25 μm. (E) Higher magnification of one SG1-treated cell expressing LC3 proteins. Scale bar is 10 μm. **(b)** LC3-expression in U-87MG cells treated with 1 μM SG-1, SG-2, or T1AM for 30 min, 4, 8, and 24 h. Data are normalized to the appropriate control values. Statistical analysis performed by student’s *t*-test. ^∗^*P* < 0.05 compared with baseline conditions; ^∗∗^*P* < 0.01 compared with baseline conditions.

As shown in **Figures 10a,b**, IF experiments revealed significant LC3-puncta formation in U-87MG exposed to 1 μM SG-1 for 4, 8, and 24 h (^∗^*P* < 0.05 and ^∗∗^*P* < 0.01 compared with baseline conditions), whereas SG-2 and T1AM treatments (1 μM) produced significant effects only at 8 and 24 h of incubation times (^∗^*P* < 0.05 and ^∗∗^*P* < 0.01 compared with baseline conditions).

**FIGURE 11:**
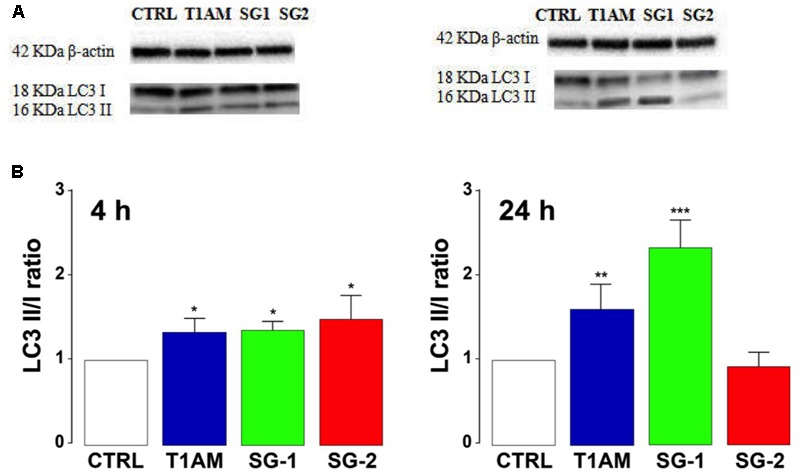
T1AM, SG-1, or SG-2 increase LC3II/LC3I ratio in U-87MG cells. **(A)** Representative bands. **(B)** Western blot quantification of LC3II/LC3I ratio. The presence of LC3 in autophagosomes and the conversion of LC3 to the lower migrating form LC3-II are used as an indicator of autophagy. Results represent mean ± SEM of three different gels. ^∗^*P* < 0.05, ^∗∗^*P* < 0.01, ^∗∗∗^*P* < 0.001 versus vehicle treated cells (Control).

As shown in **Figure 11**, Western blot analysis confirmed autophagy induction in U-87MG cells, revealing a significant LC3-II up-regulation, after 4 h treatment with all the compounds examined (^∗^*P* < 0.05 versus vehicle treated cells). After 24 h treatment only T1AM and SG-1 appeared to induce a significant LC3 II up regulation (^∗∗^*P* < 0.01 and ^∗∗∗^*P* < 0.001 versus vehicle treated cells).

**FIGURE 12:**
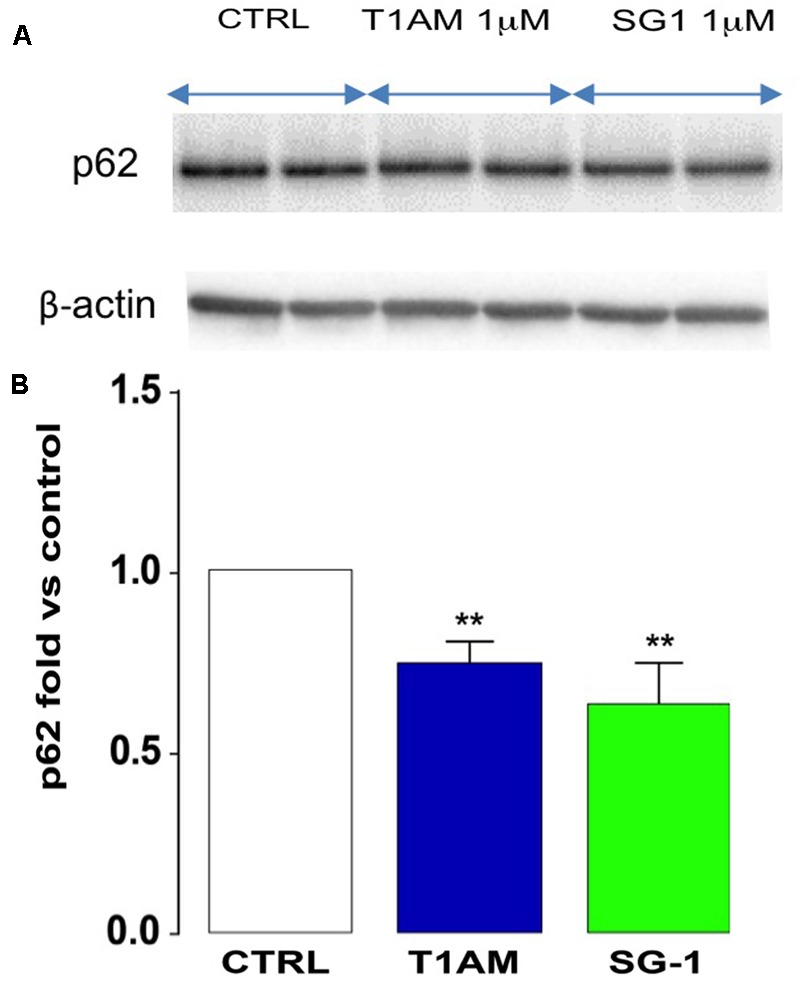
Reduced p62 expression in U-87MG cells. **(A)** Representative bands. **(B)** After 24 h, T1AM (1 μM) and SG-1 (1 μM) induced p62 degradation in U-87MG cells. Results represent mean ± SEM of three different gels. ^∗∗^*P* < 0.01 versus vehicle treated cells (Control).

Since ultrastructural studies (TEM and IF) and Western blot analysis have shown that T1AM and SG-1 are more effective than SG-2 at inducing autophagy in U-87MG cells, we next check the ability of T1AM and SG-1 to modulate the autophagic flux by examining their effect on p62 expression in U-87MG cells after 24 h incubation time. Consistently, a significant reduction of p62 expression was observed in U-87MG cells after treatment for 24 h with 1 μM T1AM and SG-1 (^∗∗^*P* < 0.01 versus vehicle treated cells) (**Figure 12**), in turn confirming their efficacy as autophagy inducers.

### T1AM, SG-1, and SG-2 Induce Autophagy via PI3K/AKT/mTOR Pathway

**FIGURE 13:**
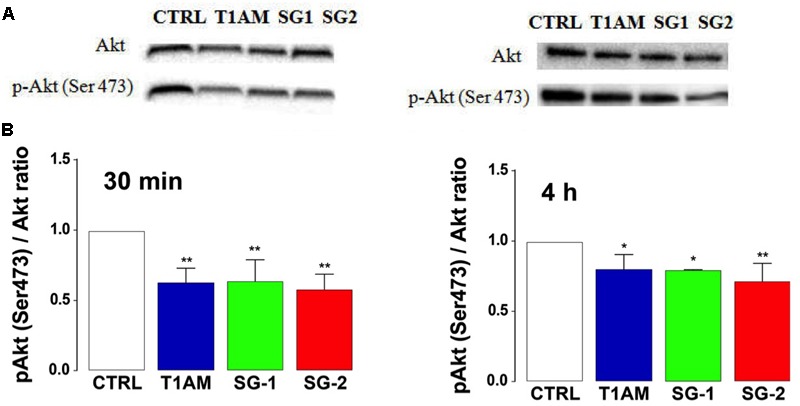
T1AM, SG-1 and SG-2 reduce the phosphorylation levels of Akt-Ser473 in U-87MG cells. **(A)** Representative bands and **(B)** Western blot quantification of pAkt/Akt ratio in U-87MG cells after treatment with 1 μM T1AM, SG-1, or SG-2 for 30 min and 4 h. Results represent mean ± SEM of three different gels. ^∗^*P* < 0.05, ^∗∗^*P* < 0.01, versus vehicle treated cells (Control).

The PI3K/AKT/mTOR signaling pathway is an important regulator of autophagy, with mTOR playing a key modulatory role (Song et al., 2005; Jung et al., 2010). Suppression of AKT has been found to decrease mTOR activity and promote autophagy (Guertin and Sabatini, 2007). Therefore, we wanted to evaluate whether the observed induction of autophagy in U-87MG cells caused by treatment with T1AM, SG-1, and SG-2 is accomplished via inhibition of the PI3K/Akt/mTOR pathway. To this aim, we analyzed the phosphorylation status of Akt in U-87MG cells treated for 30 min and 4 h with 1 μM test compounds. As shown in **Figure 13**, T1AM and thyronamine-like analogs SG-1 and SG-2 significantly reduced the phosphorylation levels of Akt-Ser 473 in U-87MG cells (^∗^*P* < 0.05, ^∗∗^*P* < 0.01, versus vehicle treated cells), suggesting that they may induce autophagy in U-87MG cells via inhibition of the phosphorylation of mTOR by the PI3K/AKT/mTOR pathway.

**FIGURE 14:**
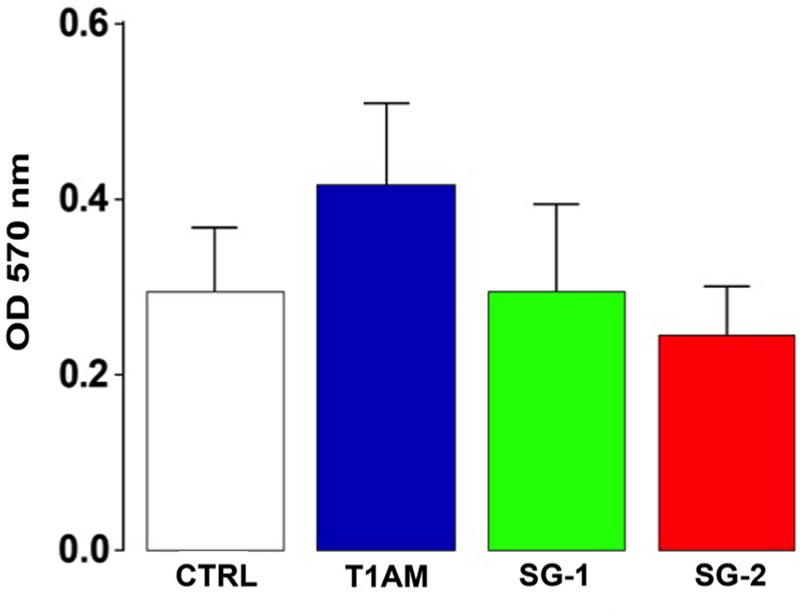
Cell viability assay. MTT assay showing U-87MG cells viability after 24 h treatment with tested compounds. Results are expressed as the mean ± SEM of 12 biological replicates.

In addition, cellular viability was determined using the MTT colorimetric assay. No significant alterations of cell viability were observed in U-87MG cells treated for 24 h with 1 μM TAAR1 agonists (i.e., T1AM, SG-1, and SG-2) as compared to vehicle (0.1% DMSO) (**Figure 14**). Absence of cytotoxicity was also confirmed by Trypan blue dye (data not shown).

## Discussion

Trace amine-associated receptor’s family (TAARs) represents a large class of receptors recently discovered that has become increasingly popular in Medicinal Chemistry as the focus of studies aimed at the discovery of new drugs (Berry et al., 2017). Among this family of receptors, TAAR1 has gained considerable interest as the target for developing therapeutic approaches against a wide range of neurodegenerative and neuropsychiatric disorders due to its widespread expression in the central nervous system, its activation by psychoactive drugs, its interrelation with the dopaminergic and serotoninergic systems, and the observation that TAAR1 knockout mice display a neurological phenotype (Miller, 2012; Lam et al., 2015).

Among TAAR1 ligands, the endogenous thyroid hormone (TH) derivative T1AM was firstly identified by Scanlan in rodent brain (Scanlan et al., 2004). Later studies demonstrated that some of its actions partly overlap to the known metabolic and neurological responses to thyroid hormone (Zucchi et al., 2014; Accorroni et al., 2017). Therefore, we might speculate that T1AM might be responsible for some effects traditionally attributed to thyroid hormone itself, in turn representing a component of thyroid hormone signaling, and consequently playing significant physiological and/or pathophysiological roles.

It is well known that thyroid hormone is fundamental for normal brain development and maintenance of optimal cognitive ability in different periods of life (Bauer et al., 2008). In adulthood, thyroid dysfunction leads to neurological and behavioral abnormalities, including memory impairment. Central hypothyroidism has been described in patients with AD (Sampaolo et al., 2005), and the analysis of various experimental models suggests that the effects on cognition rely on hippocampal alterations.

Several studies have explored the consequences of administering T1AM directly into the brain. Recently, Manni et al. (2012, 2013) reported that intracerebral T1AM administration at doses close to its physiological levels (μg/kg) evoked neurological effects improving learning capacity, decreasing pain threshold to hot stimuli, enhancing curiosity and raised plasma glycemia. Notably, the same authors observed that the effects of T1AM on memory were almost completely abolished by pretreatment of mice with clorgyline, a MAO inhibitor that prevents the oxidative deamination of T1AM, thus reducing the formation of its metabolite 3-iodothyroacetic acid (TA1). These findings suggest that TA1 might, directly or indirectly, contribute to the pharmacological effects of T1AM. Consistently, as recently shown by Musilli et al. (2014), injection in mice of equimolar doses of TA1 reproduced the same pro-learning effects induced by T1AM. Modification of memory, increase of plasma glucose and reduction of pain threshold were reported following i.c.v. injection of histamine and of selective histamine receptor agonists in rodents (Nishibori et al., 1987, 1990; Galeotti et al., 2004). Therefore, the same authors explored whether the pro-learning effects of TA1 and T1AM were modulated by histaminergic antagonists (Musilli et al., 2014; Laurino et al., 2015a,b). Their findings sustained the hypothesis that T1AM, through biotransformation into TA1, might be part of the same signaling network linking the thyroid with histamine, and that TA1 might be considered the active metabolite of T1AM that is responsible for its memory-enhancing effects. Dysregulation of the neuronal histaminergic system is a common phenomenon in different neurodegenerative disorders, including Parkinson (Anichtchik et al., 2000, 2001) and Alzheimer diseases (Trillo et al., 2013). Even though a causal relationship between thyroid, neuronal histaminergic system dysregulation and the onset of neurodegenerative diseases still need to be ascertained, maintaining a proper link between thyroid and histaminergic neurons, through the mediation of T1AM, might be critical for the preservation of behavioral circuits, including those involved in memory and learning.

The study we report here demonstrated that T1AM behaves as a memory enhancer even when injected systemically into mice at doses comparable to those previously used in central administration experiments by Manni et al. (2013). At the dosages active on memory, T1AM also proved to have an hyperalgesic effect. In addition, our pain threshold experiments confirmed that TA1, the oxidative metabolite of T1AM, plays a critical role for T1AM effect. In fact, at our settings, pretreatment with clorgyline abolished almost completely the effect on pain of T1AM administered i.p. at the highest dosage (i.e., 11 μg/kg). The same effect was also confirmed to involve the histaminergic system, as it disappeared after pretreatment with the histamine H1-receptor antagonist, pyrilamine.

Recently, the thyronamine-like TAAR1 agonist SG-2, has been shown to produce a good mimic of T1AM functional effects in rodents (Chiellini et al., 2015). Consistently, we carried out parallel experiments to explore the effects of SG-2 on memory and pain sensitivity. Our results revealed that SG-2 given i.p. to mice produce memory-enhancing and hyperalgesic effects with a potency almost comparable to that of T1AM. Notably, pretreatment with the histamine H1-receptor antagonist, pyrilamine also abolished the hyperalgesic effect elicited by SG-2. Interestingly, even though the presence of an ethoxyamino side chain should confer SG-2 a greater resistance to oxidative deamination as compared to T1AM, pretreatment with clorgyline also dampened SG-2 effects on pain sensitivity. These findings indicate that SG-2 metabolite(s) might contribute to its effects. To test this hypothesis, we explored whether SG-2 potential oxidative metabolite, namely SG-6, i.p. injected to mice (1.32, 4, and 11 μg/kg) has the capability of inducing hyperalgesia, and whether this effect is modulated by histamine. As expected, we found that SG-6, injected into mice at doses equimolar to those used for SG-2, reduced the threshold to hot stimuli with a potency even higher than SG-2. In addition, histamine appeared to be involved in SG-6 hyperalgesia, since this effect was also dampened by pretreatment with pyrilamine. Activation of ERK1/2 is a typical event induced by histamine receptor activation (Dandekar and Khan, 2011). Accordingly, increased pERK1/2 levels were found in DRG following exposure to either T1AM and SG-2. Notably, pretreatment with H_1_R antagonist pyrilamine significantly reduced ERK1/2 phosphorylation, supporting the involvement of H_1_R activation in the histamine-induced hyper-response to noxious heat (Nakagawa and Hiura, 2013).

Taken together, our results provide robust evidence that synthetic thyronamine-like analog SG-2 shares with T1AM the effectiveness on memory and pain, which seems to involve a common mechanism of action. Namely, both T1AM and SG-2 seem to rely on the action of ubiquitous enzymes MAO to produce the corresponding oxidative metabolites that are then able to activate the histaminergic system. Although our knowledge on the pharmacokinetic properties of SG-2 is still at a preliminary level, the oxidative deamination of SG-2 to generate the corresponding acid SG-6 has been observed *in vitro* using diverse human cancer cell lines, including liver cancer cells (HepG2) and glioma cells (U-87MG) (unpublished data). Even though further studies are necessary in order to prove that SG-2 generates SG-6 *in vivo*, our preliminary data support the notion that thyronamine-like analogs, such as SG-2, might represent a new option as “prodrug” candidates for the relief of memory dysfunction and disturbance of pain perception occurring in neurodegenerative diseases.

Alzheimer’s disease, accompanied by deterioration in memory and other cognitive functions, is the most frequent neurodegenerative disorder. Recent studies indicate that T1AM counteracts the effects of Aβ on LTP and restores recognition memory in a mouse model of AD (mhAPP mouse), but the underlying mechanisms are currently unknown (Accorroni and Zucchi, 2016). Recent reports have emphasized that disturbed autophagy is a contributing factor in AD (Nixon and Yang, 2011; Ghavami et al., 2014). In line with this evidence, we decided to investigate the ability of T1AM and recently developed thyronamine-like analogs SG-1 and SG-2 to induce autophagy in human glioblastoma cell lines (U-87MG). Our ultrastructural (TEM and IF) and Western blot analyses show that T1AM, as well as SG-1 and SG-2, were able to increase the LC3-II/I ratio and decreased SQSTM1(p62), indicating that they could induce autophagy. Further studies suggested that these effects might result from the inhibition of the phosphorylation of mTOR by the PI3K/AKT/mTOR pathway. Promoting cell survival while activating the autophagic process and the clearance of abnormal protein aggregates is important to neuroprotection in the treatment of neurodegenerative diseases. On the other hand, cytotoxicity or reduction of cell viability were not observed in U-87MG cells after treatment with our test compounds for 24 h. Ultimately, we provided preliminary evidence that T1AM and selected thyronamine-like TAAR1 agonists can induce autophagy. Even though at a low level, TAAR1 is expressed in U-87MG cells (Hauser and Knapp, 2014). Ongoing experiments are examining the role of TAAR1 activation in autophagy induction by pretreatment of U-87MG cells with EPPTB, a specific TAAR1 antagonist (Bradaia et al., 2009).

## Conclusion

Our data displayed that T1AM and thyronamine-like TAAR1 agonists SG-1 and SG-2 have neuroprotective properties, which also involve the induction of autophagy. This novel aspect requires further investigation. Future *in vivo* studies will be carried out to ascertain whether T1AM and TAAR1 agonist administration in mice may also be able to induce autophagy, thus providing a more conclusive evidence for the connections between memory enhancement and induction of autophagy. In addition, to confirm the positive effects on memory retention, further experiments will be performed in animal models for impaired learning processes.

Notably, neurodegenerative diseases are multifactorial debilitating disorders involving multiple pathways that, in addition to protein misfolding and aggregation, are characterized by several metabolic changes, such as mitochondrial dysfunction, oxidative stress, and phosphorylation impairment, all occurring concurrently (Nesi et al., 2017). Because of these multifactorial aspects and complexity, multi-target directed ligand (MTDL) design and discovery has emerged as a possible strategy for the treatment of neurodegenerative disorders (Saba Sheikh et al., 2013). In line with this evidence, we can speculate that the poly-pharmacological and pharmacokinetic properties described in good detail for T1AM, and rapidly emerging also for thyronamine-like analogs, SG-1 and SG-2, may provide a novel pleiotropic therapeutic approach for the treatment of neurodegenerative disorders still lacking an effective therapy.

## Author Contributions

GC, RZ, FF, and LRa designed and directed the project. LB, AL, MS, PL, AS, FB, and LRo carried out the experiments and analyzed the data. SS and SR prepared all the SG-compounds tested in the study. SR also contributed to design the project. All authors discussed the results and contributed to the final manuscript. GC wrote the manuscript.

## Conflict of Interest Statement

The authors declare that the research was conducted in the absence of any commercial or financial relationships that could be construed as a potential conflict of interest.
